# *Bhageerath*-H: A homology/*ab initio *hybrid server for predicting tertiary structures of monomeric soluble proteins

**DOI:** 10.1186/1471-2105-15-S16-S7

**Published:** 2014-12-08

**Authors:** B Jayaram, Priyanka Dhingra, Avinash Mishra, Rahul Kaushik, Goutam Mukherjee, Ankita Singh, Shashank Shekhar

**Affiliations:** 1 Department of Chemistry, Indian Institute of Technology, Hauz Khas, New Delhi-110016, India; 2 Supercomputing Facility for Bioinformatics & Computational Biology, Indian Institute of Technology, Hauz Khas, New Delhi-110016, India; 3 Kusuma School of Biological Sciences, Indian Institute of Technology, Hauz Khas, New Delhi-110016, India

**Keywords:** Protein tertiary structure prediction, *ab initio*, homology, RMSD, protein folding

## Abstract

**Background:**

The advent of human genome sequencing project has led to a spurt in the number of protein sequences in the databanks. Success of structure based drug discovery severely hinges on the availability of structures. Despite significant progresses in the area of experimental protein structure determination, the sequence-structure gap is continually widening. Data driven homology based computational methods have proved successful in predicting tertiary structures for sequences sharing medium to high sequence similarities. With dwindling similarities of query sequences, advanced homology/ *ab initio *hybrid approaches are being explored to solve structure prediction problem. Here we describe *Bhageerath*-H, a homology/ *ab initio *hybrid software/server for predicting protein tertiary structures with advancing drug design attempts as one of the goals.

**Results:**

*Bhageerath*-H web-server was validated on 75 CASP10 targets which showed TM-scores ≥0.5 in 91% of the cases and Cα RMSDs ≤5Å from the native in 58% of the targets, which is well above the CASP10 water mark. Comparison with some leading servers demonstrated the uniqueness of the hybrid methodology in effectively sampling conformational space, scoring best decoys and refining low resolution models to high and medium resolution.

**Conclusion:**

*Bhageerath*-H methodology is web enabled for the scientific community as a freely accessible web server. The methodology is fielded in the on-going CASP11 experiment.

## Background

"The native conformation of a protein is determined by the totality of interatomic interactions and hence, by the amino acid sequence, in a given environment" (Nobel Lecture, Christian B. Anfinsen, December 11, 1972). According to Anfinsen's protein folding hypothesis, a protein's native structure is determined by its amino acid sequence which drives protein into its minimum Gibbs energy state [1]. This hypothesis evolved as a basic tenet for protein structure prediction algorithms (PSPAs). However limited understanding of net balance of forces involved in protein folding creates deficiencies in various proposed PSPAs. One of the early efforts in solving protein folding problem was driven by thermodynamic calculations, which incorporate searching algorithms to investigate a conformation that corresponds to minimum free energy [2]. Here the large number of degrees of freedom of a protein gives rise to innumerable conformations, an enumeration of which is practically impossible. This despite, proteins fold rapidly into their native structure in milliseconds to seconds time scales implying that a brute force enumeration of all possible conformations may not be required as implicit in Levinthal's Paradox [3]. The fact that sequence introduces local structural bias, narrows down the accessible conformational space and introduces local as well as long range interactions, suggesting a halfway solution to the paradox [4-7]. As a result, PSPAs need two key components: (a) a rapid computational algorithm for protein conformational search and (b) an accurate scoring function to capture the best available conformation. The first component involves use of different physics based as well as knowledge based approaches for extensive sampling of the vast conformational space [8,9]. Physics based sampling methods include use of Monte Carlo (MC) methods [10-15], Genetic algorithms [16], molecular dynamics simulations (MD) [17,18], simulated annealing [19,20], replica-exchange MC or MD and local enhanced sampling [21-23]. Knowledge based methods use information from the solved protein structures and knowledge based potentials for sampling protein conformational space [24].

Homology modeling [25-30] and fold recognition/ threading methods [31-35] are knowledge based approaches, which are routinely used to generate reliable models for proteins with overall fold topology similar to an available template in the protein databases. Query protein with no sequence and structural similarity are modeled from scratch using physics based/ *ab initio *approaches. The success of *ab initio *or physics-based sampling methods is limited by lack of accurate energy functions [36,37], heavy computational requirements, force field errors [38-40] and protein size, while knowledge based approaches are limited by sequence similarity and evolutionary relationships [41-43]. A popular trend in protein conformational sampling is the fragment assembly method, which uses parts of known protein or protein fragments to generate a structure of the target. After conformational sampling, the next immediate concern is to capture the best available structure by means of a scoring function [44-56]. These functions combine chemical, physical, geometrical and energetic constraints to capture native or near native models [57,58].

A thorough literature survey reveals that the available protein structure prediction algorithms are based on methods such as (a) homology modeling, (b) fold recognition, (c) *ab initio *and (d) hybrid [59,60]. Different software/tools are available in the public domain based on these computational approaches and are evaluated every two years during the Critical Assessment of techniques for protein structure prediction (CASP experiments) [61]. Recent CASP experiments have shown significant progress by hybrid approaches, which combine homology, *ab initio *along with atomic level model refinements for protein structure prediction [62]. This article describes *Bhageerath*-H, a homology/ *ab initio *hybrid software for predicting tertiary structure of monomeric proteins. *Bhageerath*-H makes use of *Bhageerath*-H Strgen algorithm [63] for extensive sampling of the protein fold space and generates a large basket of decoys containing near-native protein conformations, which are further supplemented by a chemical logic based alignment scheme and then clustered to eliminate non-unique redundant structures. These are then screened by a physico-chemical scoring metric (pcSM) and assessed for their quality. The selected models are refined via a unique and effective quantum mechanics based loop bond angle optimization method, which drives the selected models further close to the native topology. *Bhageerath*-H automated pipeline is freely available to the scientific community across the world via http://www.scfbio-iitd.res.in/bhageerath/bhageerath_h.jsp.

## Methodology

**Figure 1:**
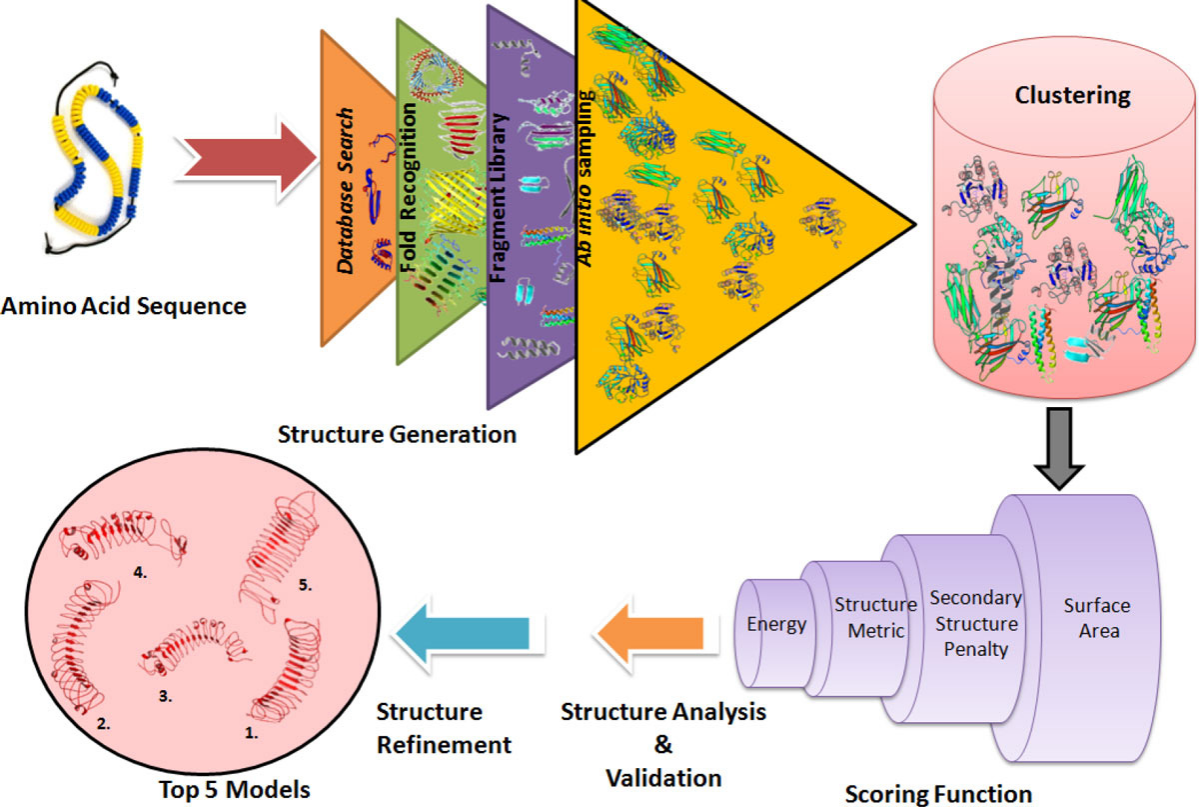
**Diagrammatic representation of *Bhageerath*-H protocol**.

*Bhageerath*-H software suite for protein tertiary structure prediction narrows down the conformational search space and predicts five probable near native candidate structures for an input amino acid sequence. The software comprises seven computational modules which work in conduit and together form an automated pipeline. Figure 1 shows a diagrammatic representation of *Bhageerath*-H software suite. Following sections discuss each module of the automated pipeline.

### (A) Bhageerath-H Strgen for candidate structures

The first step in the pipeline involves generation of a large pool of full length decoys. In the proposed protein structure prediction pipeline, *Bhageerath*-H Strgen algorithm for protein conformational sampling [63] is the first module. The module takes as input protein amino acid sequence and provides as output a large pool of decoys. A revised and improved version of structure generation algorithm is incorporated in the *Bhageerath*-H software suite. *Bhageerath*-H Strgen makes use of the current sequence and structural database knowledge along with *Bhageerath ab initio *folding [64,65] in order to effectively search the fold space for an input protein sequence. It starts with amino acid sequence, followed by secondary structure prediction and BLAST [66] search for sequence based homologs. In addition, it also searches for distant analogs and structural homologs using tools such as pGenthreader [67,68], ffas [69,70], spark-x [71] and HHSearch [72]. A new addition to this methodology is a chemical logic based [73] procedure for template selection followed by alignment generation. It utilizes amino acid chemical properties such as hydrogen bond donor, conformational flexibility, shape and size of side chains for generating an amino acid substitution scoring matrix. This scoring matrix is used for template selection as well as template-target alignment generation. The matrix helps in selecting distant homologs, which are generally missed during a normal database search. The templates and template-target alignments are used for modeling fragments of varying length via Modeller [74,75]. Modeled fragments are then screened for missing links with no available templates. These missing stretches are generated using *Bhageerath ab initio *modeling method [65,76,77]. All the incomplete protein fragments are patched in order to generate full-length models, which are energy scored and top 5 lowest energy decoys are sent for *Bhageerath abintio *loop sampling. The newly sampled structures are added to the growing pool of full length protein decoys. The output of the first step is a large pool of protein decoys. The average size of the decoy pool is on the order of 10^4^-10^5 ^structures.

*Bhageerath*-H Strgen module includes locally installed copies of Psipred, BLAST, PFAM [78], SCOP [79,80], nr [81], pdb database [82]http://www.pdb.org/pdb/home/home.do, HHSearch, Spark-X, pGenthreader, ffas and modeller. The scalable *Bhageerath*-H Strgen algorithm is currently configured to utilize 64 processors of Linux Cluster. Programs are written in C++, MPI language and involve use of linux shell scripting. Average time taken for *Bhageerath*-H Strgen run is 1-2hrs. This first module of the *Bhageerath*-H pipeline generates a large pool of decoys which needs to be further filtered, processed and refined. We would like to note that *Bhageerath*-H software is not just limited to *Bhageerath*-H Strgen an already published algorithm. *Bhageerath*-H Strgen is a protein decoy generation program which is the first module here. After protein decoy generation, protein decoy selection and refinement are the other two very important steps in protein structure prediction pipeline. In *Bhageerath*-H software modules 2-5 are dedicated for decoy clustering, selection and refinement, which are not included in *Bhageerath*-H Strgen. Output from this module is submitted for clustering in the next step.

### (B) Clustering

Recurring structural models sampled in the previous step are clustered using K-means clustering algorithm. The main aim of this step is to retain a single representative structure of each unique topology. MMTSB [83] toolkit's *k-clust *is used to perform clustering. The tool requires list of protein decoys to cluster. Following command was executed:

kclust -mode rmsd -cdist -heavy -lsqfit -radius 1.0 -maxerr 1 pdblist>cluster_file

This command gives as an output a cluster file, which contains the centroid in the pdb format along with the members of each centroid and the root mean square deviation (rmsd) distance of each member from the centroid. The centroids themselves are mathematical constructs and convey no information, but utilizing rmsd information one lowest rmsd member from each cluster is picked [83]. To overcome the time limitation, clustering is performed in a parallel mode. The output of K-mean clustering is a set of decoys, which are unique, non-recurring and contain near-native structural models. This set of decoys containing near-native models is submitted for physico-chemical scoring in the third step.

### (C) Scoring based on a physico-chemical metric

The third step in the *Bhageerath*-H pathway involves the use of a robust metric that combines chemical, physical, geometrical and energetic constraints known to show universalities among native protein structures. The physico-chemical scoring metric (pcSM) consists of different parameters, which include (a) **P: **Secondary structure penalty, (b) **M: **Euclidean distance, (c) **A1-A4: **Surface areas and (d) **E: **Empirical potential energy functions. The scoring function calculates a final cumulative score (CS), which comprises each of these parameters.

$$CS=c_{A1}A1+c_{A2}A2+c_{A3}A3+c_{A4}A4+c_{pmax}\left(P_{H},P_{S}\right)+c_{M1}M1$$

where A1 is the fractional area of exposed non-polar residues, A2 is the fractional area of exposed non polar part of residues, A3 is the weighted exposed area, A4 is the total surface area, PH and Ps are secondary structure penalties for helix and sheet respectively, M1 is Euclidean distance. The prefix "c" for each parameter in the above equation refers to its optimized coefficient. c_A1 _= 10, c_A2 _= 0.1, c_A3 _= 0.00001, c_A4 _= 0.001, c_M1 _= 0.001, c_p _= 0.15(P_H_) and 0.21(P_S_).

In order to get the top 10 structures, each of the seven parameters are evaluated for all the clustered decoys and a short energy minimization is performed to remove steric clashes. For the given input decoy pool, pcSM gives as an output top 10 ranked native-like candidates structures. pcSM algorithm runs in parallel mode and utilizes 64 processors. On an average, time taken for scoring varies from 2 to 3 hours. The top 10 pcSM ranked models are submitted for protein structure analysis and validation in the next step.

### (D) Protein Structure Analysis and Validation (PROTSAV) based ranking

PROTSAV is a protein structure quality assessment meta-server (manuscript under preparation). Currently, it comprises six tools namely Procheck [84], Verify-3D [85], ERRAT [86], Naccess [87], PROSA [88] and dDFIRE [89], for quality assessment of protein structures. PROTSAV generates an overall protein quality score, which is a summation of scores predicted by individual modules. High PROTSAV values reflect poor structure quality of query protein and low values close to zero represent good quality of query protein structure. Run time for this module is 40-45 seconds. In this step, pcSM selected top 10 protein models are analysed and ranked. The top ranked model is submitted for QM based loop bond angle refinement in the next step.

### (E) Quantum mechanics (PM6) based loop bond angle optimization

Quantum mechanics (PM6) based loop bond angle optimization (manuscript in preparation) takes topmost PROTSAV selected model as an input, optimizes loop bond angles and performs *ab initio *loop sampling [66]. The small pool of decoys generated in the process is side chain optimized using Scwrl4. Scwrl4 is a program for prediction of protein side chain conformation [90]. Scwrl4 uses latest backbone-dependent library to provide rotamer frequency, dihedral angles and variances. The side chain optimized decoys are further energy minimized (SD = 500, CG = 500) using sander module of AMBER10 software [91].

These optimized and energy minimized refinement generated decoys are scored using pcSM and the top 10 ranked QM refined models are passed to next step.

### (F) Final ranking

Input to this step is top 10 pcSM ranked QM refined models from step (E) and top 5 PROTSAV ranked models from the step (D). PROTSAV ranked models are side chain optimized and energy minimized before final ranking. The selected 15 models are re-ranked using pcSM and the top 5 are given to the user as an output.

**Figure 2:**
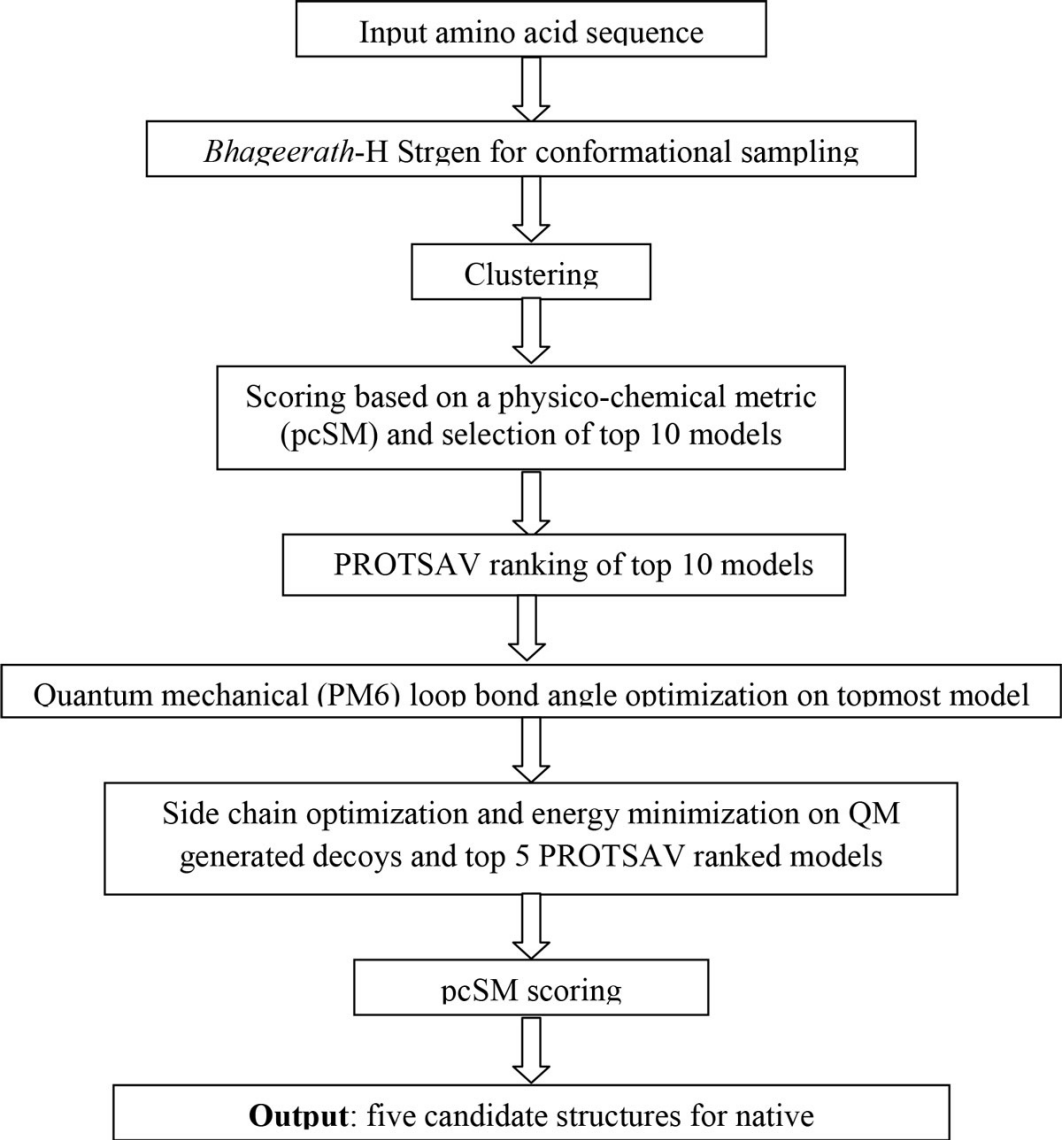
**Flowchart of *Bhageerath*-H software**.

The *Bhageerath*-H protocol is a careful combination of different algorithms which are configured to work in conduit. Starting from *Bhageerath*-H Strgen followed by clustering, pcSM scoring, PROTSAV and QM refinement each module has its own importance and role in providing the user, near-native candidate structures as final output. The software takes protein amino acid sequence as input and provides a user as output five native-like candidate structures. Figure 2 shows the flow chart of *Bhageerath*-H software suite.

## Results and Discussion

### Validation of *Bhageerath*-H software suite

*Bhageerath*-H automated pipeline was thoroughly tested and validated on the benchmark CASP10 dataset. Each CASP experiment reveals the state of the art in the field of protein structure prediction. About75 CASP10 targets of varying size and complexity were considered here for the analysis. To begin with the assessment, CASP-like conditions were mimicked, which means the native and near-native homologs were excluded during structure prediction. Any template released later than the first CASP10 server target i.e. fifth May of 2012 was not considered. For structure assessment an automated pipeline was developed. For each CASP10 target, sequence was extracted from the native structure. Then predicted structure sequence and the native sequence were aligned using ClustalW [92]. Residues with missing coordinates were removed from the predictions in order to make the sequence of the two structures match exactly. The native and the *Bhageerath*-H generated final five models were compared based on the widely used criteria of Cα root mean square deviation (Cα RMSD) and Template modeling score (TM-score). Cα RMSD is a global indicator of structural identity, while TM-score identifies local substructures and evaluates local identity. TM-score refers to template modeling score. TM-score is considered as a quantitative measure for classification of protein topology. A TM-score > 0.5 signifies that protein pairs share same fold whereas a TM-score < 0.5 are mostly not of the same fold and a TM-score of 0.17 indicates random prediction [93,94].

#### (A) Bhageerath-H performance on 75 CASP10 targets

**Figure 3:**
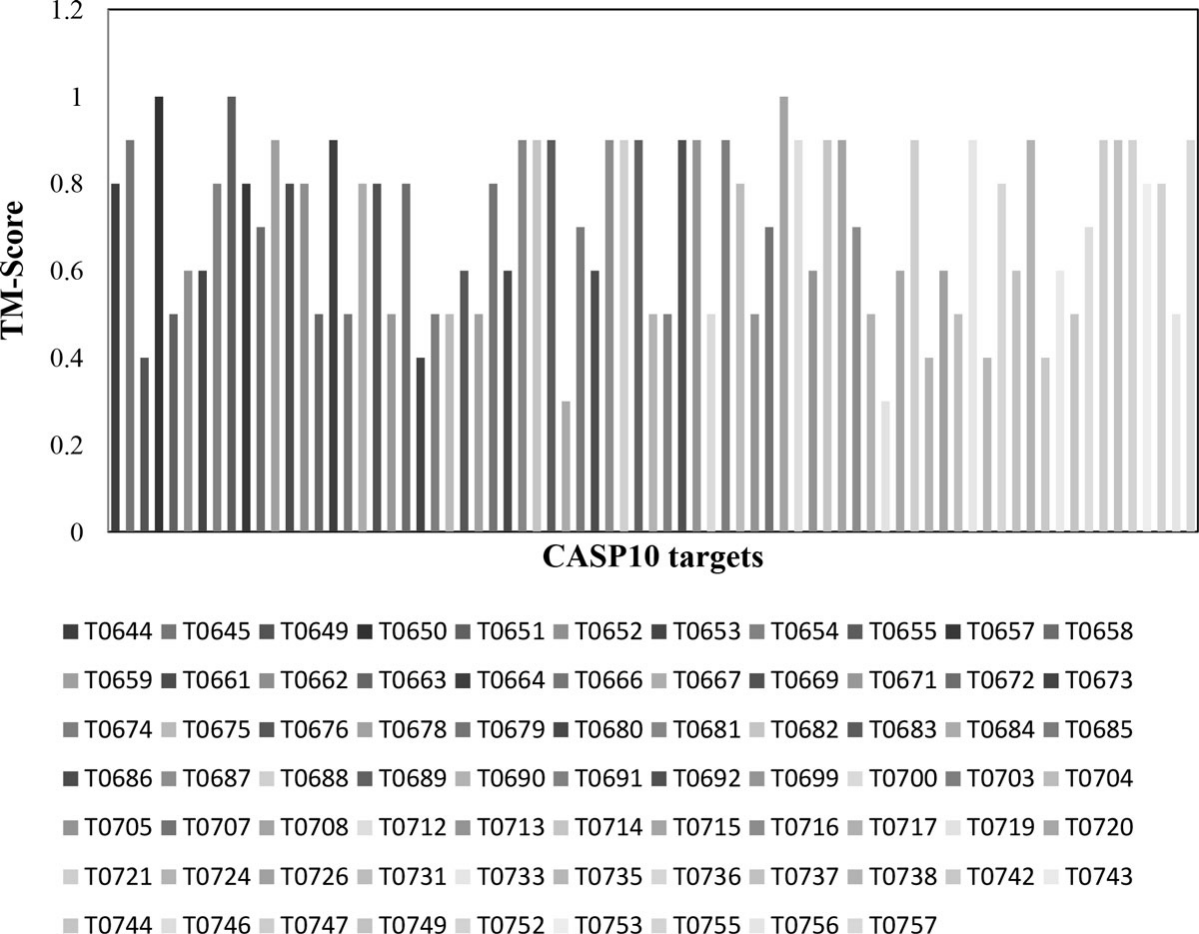
**TM-score distribution of 75 CASP10 targets**.

**Figure 4:**
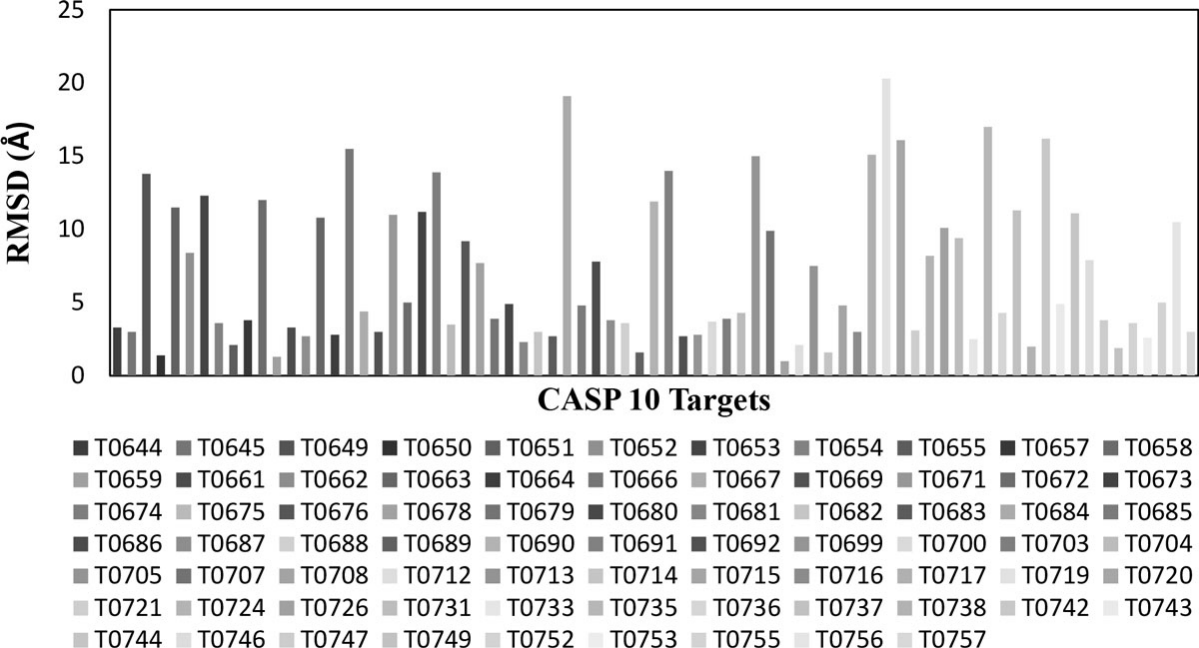
**Cα RMSD distribution of 75 CASP10 targets**.

*Bhageerath*-H was validated on 75 CASP10 targets. Cα RMSDs and TM-scores of final five *Bhageerath*-H predictions from the native were calculated. In 68 out of 75 systems i.e. in 91% of the cases *Bhageerath*-H predicted model has a TM-score ≥0.5, while in 44 targets i.e. in 59% of the cases *Bhageerath*-H was able to predict a model in top 5 having a Cα RMSD from the native ≤5.0Å (Additional File 1). Figure 3 shows the TM-score distribution and Figure 4 shows the Cα RMSD distribution of all the75 targets.

##### Comparison of Bhageerath-H performance with BAKER-ROSETTA, Quark and MULTICOM-CLUSTER

**Figure 5:**
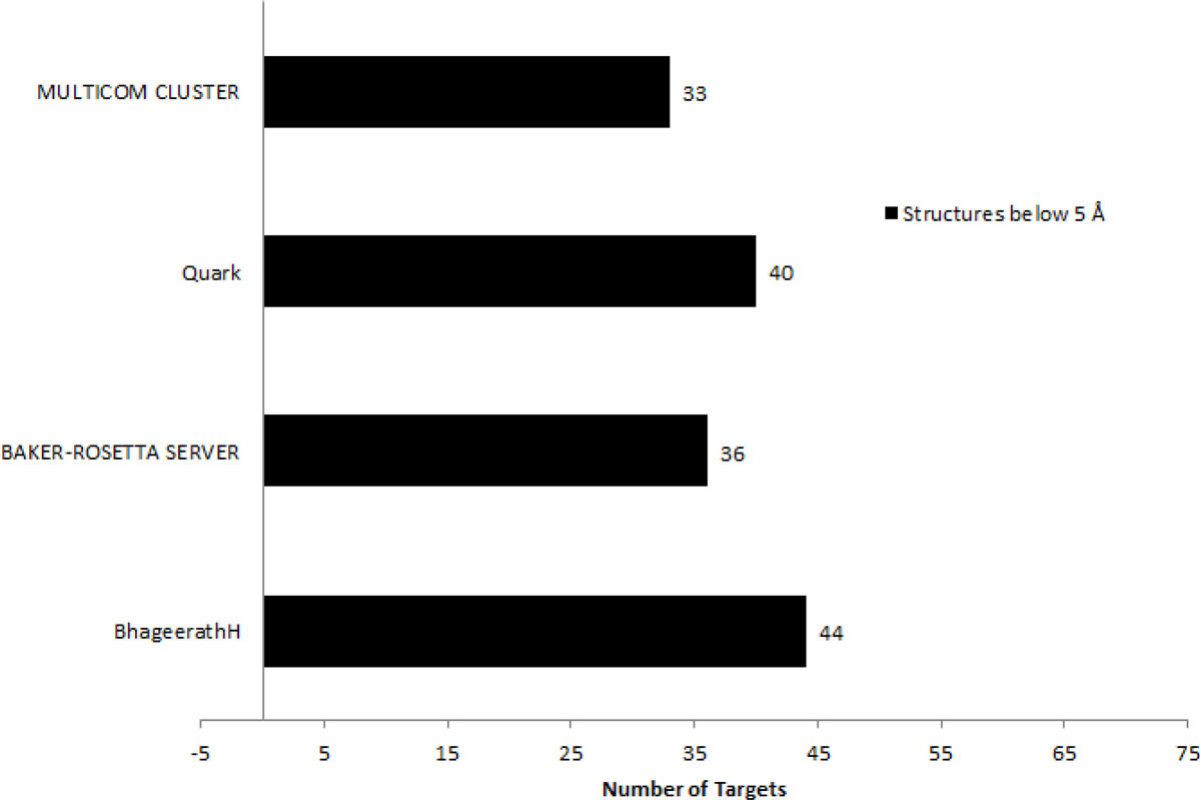
**A comparative study of 75 CASP 10 target predictions under RMSD cut-off of 5 Å by *Bhageerath*-H, BAKER-ROSETTA, Quark and MULTICOM-CLUSTER server**.

For comparative analyses, we considered three state-of-the-art servers for protein tertiary structure prediction. Predictions submitted by BAKER-ROSETTA [95], Quark [96] and MULTICOM-CLUSTER [97] during CASP10 [62] experiment were used. Their submitted five predictions were downloaded from the CASP10 website http://www.predictioncenter.org/casp10/index.cgi and analyzed using the automated evaluation pipeline described above. The minimum RMSD obtained among the five submitted models was considered. In 36 cases, BAKER-ROSETTA server submitted a model among five predictions having Cα RMSD from the native ≤5.0Å. Quark submitted 40 predictions among 75 under the Cα RMSD cutoff of 5.0Å, whereas MULTICOM-CLUSTER succeeded in 33 cases. In comparison to these three servers, *Bhageerath*-H server was successful in 44 cases i.e. in 59% of the cases, this server was able to propose a model in top 5 having a Cα RMSD from the native ≤5.0Å (Figure 5).

CASP organizers assign a unique target id to each protein fielded in the CASP experiment. While validating and comparing performance of *Bhageerath*-H software on 75 CASP10 targets, we have closely analyzed some of the CASP10 target proteins in which *Bhageerath*-H outperformed other three servers under consideration. A brief description of the biological role of the targets T0655, T0672, T0675, T0700, T0716, T0736, T0747, T0755, T0669, T0713, T0686, T0724 is given in Additional File 2.

**Figure 6:**
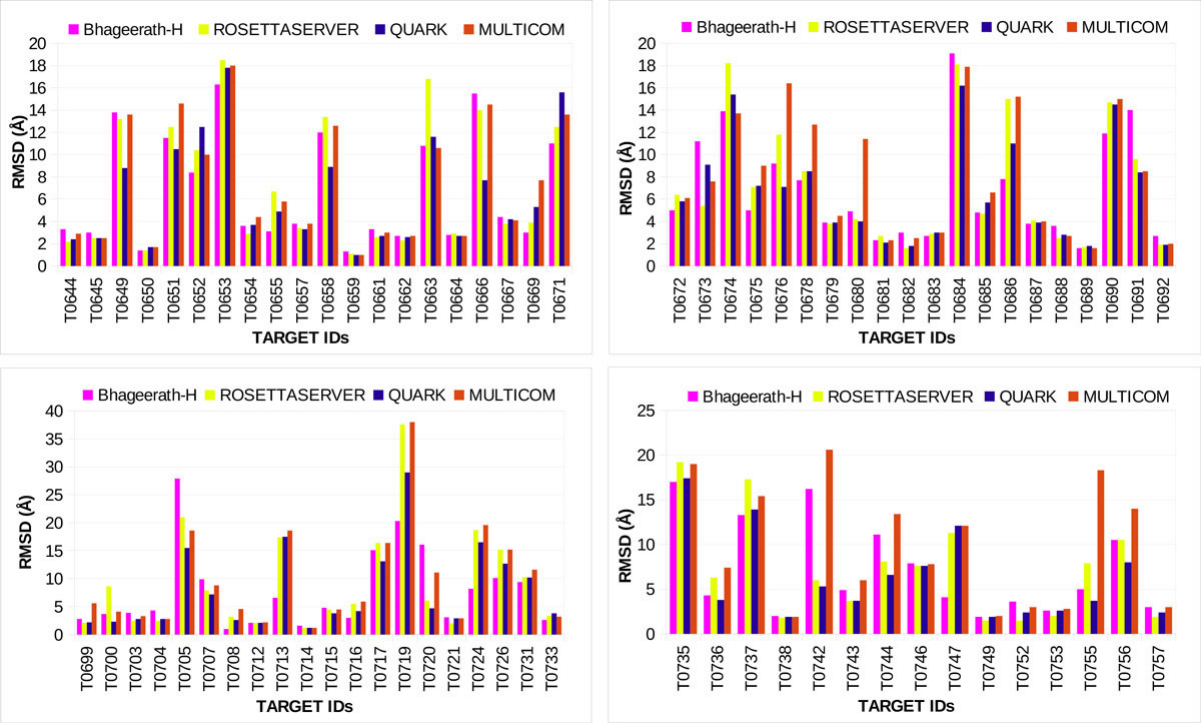
**Comparison of *Bhageerath*-H software suite with BAKER-ROSETTA server, Quark server and MULTICOM-CLUSTER server for 75 CASP10 targets**.

For targets T0655, T0672, T0675, T0700, T0716, T0736, T0747, T0755 *Bhageerath*-H outperformed BAKER-ROSETTA server. It predicted a structure in top 5 within the defined Cα RMSD cutoff (≤5.0 Å). In case of Quark, *Bhageerath*-H exceeded in 6 cases T0669, T0672, T0675, T0685, T0716, T0747, while *Bhageerath*-H was successful in 11 cases when compared with MULTICOM-CLUSTER. For targets T0655, T0672, T0675, T0716, T0747, *Bhageerath*-H achieved high prediction accuracy than all the three servers (Figure 6).

A close inspection of the reason for better performance of *Bhageerath*-H revealed that for targets such as T0675, T0672, T0669, T0716, T0736, T0700 it was *Bhageerath*-H Strgen patching module as well as *ab initio *loop sampling which generated a low RMSD near-native structure. In systems T0655, T0747, the low RMSD sampled structure is due to the amino acid chemical logic based scoring matrix. The amino acid substitution scoring matrix is a new addition to *Bhageerath*-H Strgen methodology and performs a very thorough search of the database for homologs based on amino acids chemical properties. This matrix helped in template search and alignment generation especially in targets T0655 and T0747, where most other servers failed to predict a low RMSD structure. It identified correct templates and generated better target-template alignments, which resulted in high quality near-native structural models for proteins with low sequence similarity. In cases where a full length template is unavailable, the matrix helped in generating high quality alignments for short sequence fragments. Other than amino acid chemical logic based scoring matrix the major contributor for better performance of *Bhageerath*-H software is *abinitio *loop sampling. Loops are the most flexible parts of a protein structure involved in molecular recognition. Correct modeling of loops has always been a challenge. *Ab initio *loop sampling module helped in systematic and thorough sampling of the loop conformation space and generated low RMSD models. CASP 10 targets where *Bhageerath*-H outperformed other participating servers were mainly modeled through chemical logic and *ab-initio *loop sampling.

Other than above specified targets, *Bhageerath*-H's performance is noteworthy for targets T0713, T0686 and T0724 when compared to the other three servers under consideration. Though high quality *Bhageerath*-H models were not predicted, these targets need special attention and discussion. These three targets are described below as case studies for illustration of *Bhageerath*-H performance.

(i) **Target T0713**: This target is a hypothetical protein from *Eubacterium ventriosum *having PDB: 4H09 and 739 amino acid residues. It has four leucine rich repeats domains which take solenoid shape in protein structure. These domains help protein to interact with its complementary protein partner. *Bhageerath*-H sampled a lowest RMSD structure of 8.91Å in pool of trial structures. After clustering and pcSM decoy selection the lowest RMSD model in top 10 was 9.80Å. The topmost PROTSAV selected model was given to QM based structure refinement. QM refined the input model and generated a decoy in the small pool having 6.61Å Cα RMSD from the native. It is due to the bond angle optimization which assisted in a better conformational sampling and a lower RMSD decoy, which was picked by pcSM during final five ranking. *Bhageerath*-H successfully modeled and picked a structure in the top five having leucine repeat domain similar to the native structure. The domain form horseshoe shape reflects its biological activity.

(ii) **Target T0686**: This target is a sporozite surface protein of *plasmodium vivax*, one of the causative agents for malarial disease. It is also called TRAP (thrombospondin repeat anonymous protein) which mediates the invasion of mosquitoes and vertebrates host cells in malaria. TRAP protein has two functional domains (i) TSP (thrombospondin type I) and (ii) VWA (von willebrand factor type A) that are responsible for cell adhesion. *Bhageerath*-H Strgen generated a 7.41Å RMSD structure which was retained post clustering. pcSM and PROTSAV picked an 8.13Å structure which was submitted for QM based refinement. The final lowest RMSD model in top 5 is 7.75Å, which is a much better prediction in comparison to other server predictions. Model structure closely superimposes with VWA domain of native crystal structure (PDB: 4QHO) protein while there are a few anomalies in TSP domain. VWA domain is mainly responsible for protein's biological activity and covers a stretch of ~180 amino acids. TSP is a shorter domain (∼40 amino acids). The final ranked *Bhageerath*-H modelled structure missed an extended β-sheet, which resulted in a high RMSD of the prediction from the native.

(iii) **Target T0724**: This target is a hypothetical uncharacterized protein from *bacteroides vulgates *having PDB: 4FMR. It has only one characterized functional domain i.e DNA binding. QM based structure refinement assisted in better conformational sampling and in generating a near-native decoy. A brief biological description of the studied targets is given in the Additional File 2.

In a nut shell, major reasons behind the ability of *Bhageerath*-H to predict lower RMSD near-native models are firstly exhaustive sampling technique. *Bhageerath*-H Strgen and the newly developed amino acid chemical logic based scoring matrix help in a thorough search of template and protein conformational space, ensuring generation of near-native models in maximum instances. Secondly, it is the pcSM scoring function which cherry picks these native-like candidates with 93% accuracy. Apart from these two major modules, it is the PROTSAV structure analysis which ranks models accordingly and submits for QM refinement. Finally, QM based refinement protocol facilitates in going one step ahead and improves prediction accuracy.

#### (B) Assessment of individual modules of Bhageerath-H pipeline

To comprehend the potential of individual modules of *Bhageerath*-H automated pipeline, we further analyzed 7 targets where *Bhageerath*-H outperformed all the three servers. Table 1 details the output of individual modules of *Bhageerath*-H i.e *Bhageerath*-H Strgen, clustering, pcSM scoring, PROTSAV ranking and final output. Table 1 column 3 contains the result of module 1, *Bhageerath*-H Strgen. It shows the lowest Cα RMSD sampled in the decoy pool. Column 4 shows the size of the decoy pool. Column 5 has Cα RMSD result for module 2, clustering. It contains information of the lowest RMSD structure in the decoy pool after clustering. Column 6 represents the size of the decoy pool post clustering. Column 7 contains the result for module 3, pcSM scoring. It shows the lowest Cα RMSD among the top 10 pcSM ranked decoys. Column 8 has results of module 4, PROTSAV ranking. The Cα RMSD of topmost PROTSAV ranked model. The last column has the final prediction results of *Bhageerath*-H pipeline, the lowest Cα RMSD among final five *Bhageerath*-H predictions for the given target.

**Table 1:** Assessment of individual modules of *Bhageerath*-H pipeline for 7 CASP10 targets.

S.No	Target	Lowest Cα RMSD structure in *Bhageerath*-H Strgen sampled decoy pool	Number of decoys generated	Lowest Cα RMSD structure post K-mean clustering	Number of filtered decoy	Lowest Cα RMSD structure among top10 pcSM ranked decoys	Cα RMSD of the topmost SAVPRO ranked model	Lowest Cα RMSD among final five *Bhageerath*-H predictions
1.	T0655	2.98	77826	3.13	19742	3.13	3.13	3.13

2	T0669	2.85	20002	2.85	7634	2.85	2.85	2.85

3	T0675	4.90	108131	4.95	31104	5.05	5.1	4.93

4	T0716	2.97	109056	2.97	11708	2.97	3.06	2.97

5	T0733	2.54	105124	2.54	14460	2.54	2.65	2.59

6	T0747	4.11	60584	4.11	17874	4.11	4.11	4.11

7	T0672	5.00	116786	5.00	26806	5.00	5.09	5.00

**Figure 7:**
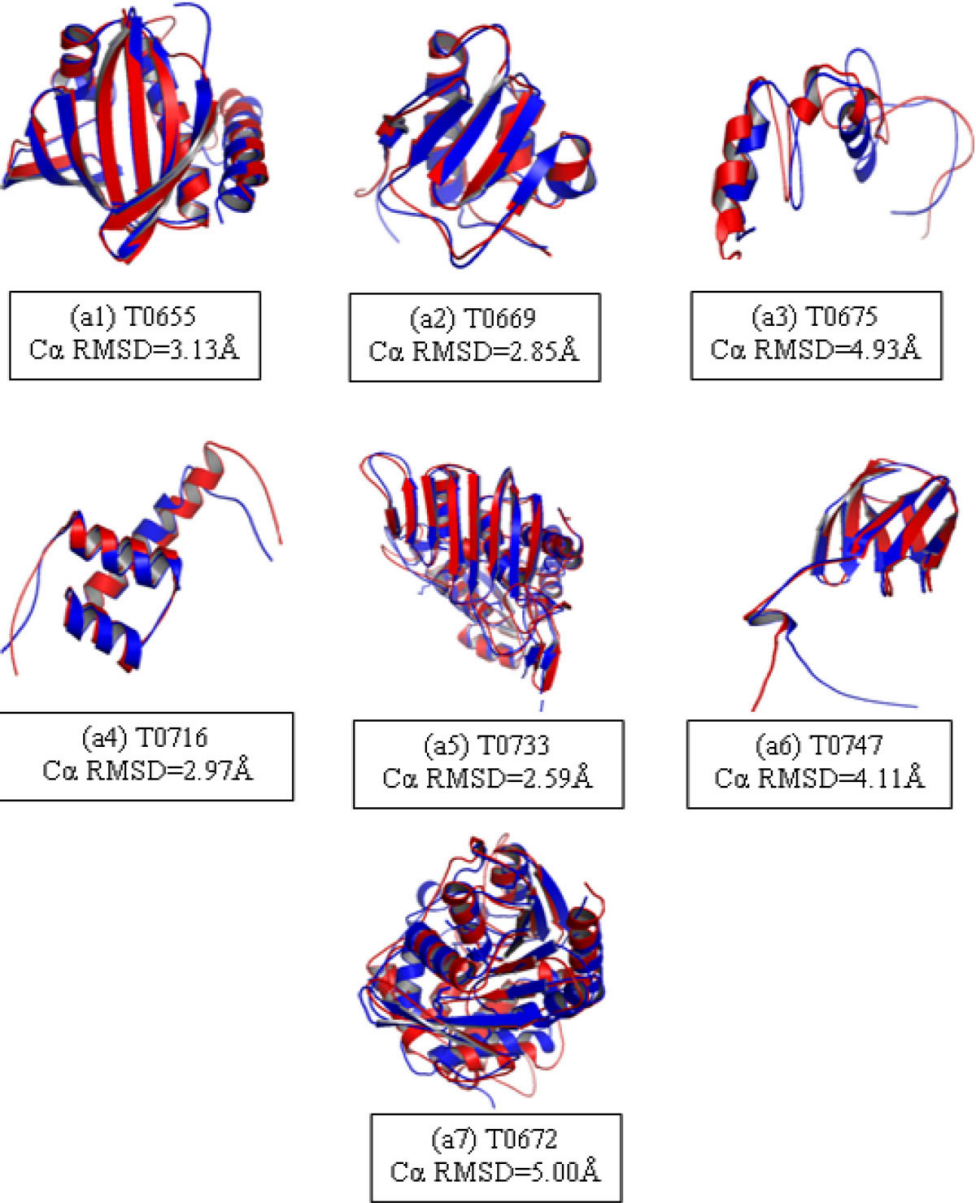
**(a1-a7): A superimposition of best *Bhageerath*-H predicted model with native for 7 targets**. *Bhageerath*-H predicted model is in red and native is in blue.

As discussed earlier the backbone of any protein tertiary structure prediction software/tool is its protein conformational sampling module. Unless a near-native decoy is sampled/generated, it is impossible to attain high prediction accuracy. In Table 1 for all the 7 targets near-native decoys (Cα RMSD ≤5.0Å) were present in *Bhageerath*-H Strgen sampled decoy pool. These decoys were retained post K-mean clustering. While filtering bad decoys from good ones, it is extremely important to retain the sampled near-native decoys in the smaller basket. As can be seen from Table 1 clustering was able to reduce the basket size while retaining good structures. Second major module of prediction pipeline is scoring. pcSM scoring function has successfully picked the best decoys in top10 except in the case of T0655. PROTSAV has further assisted in ranking the best model (lowest Cα RMSD) as topmost model in 5 cases. In 2 cases we missed out the lowest RMSD sampled decoy in final ranking but successfully selected a ≤5 Å in final predicted output. The last column shows the final prediction results of *Bhageerath*-H pipeline. Figure 7(a1-a7) shows superimposition of lowest Cα RMSD *Bhageerath*-H predicted models with the corresponding natives.

#### (D) Quality assessment of Bhageerath-H predictions

Finally, the quality of *Bhageerath*-H predictions was assessed based on Molprobity score [98]. Molprobity score evaluates the stereochemistry of input structure. Online Molprobity server http://molprobity.biochem.duke.edu was used for score calculation. Additional File 3 shows the Molprobity score of the best *Bhageerath*-H predictions. Best refers to the lowest Cα RMSD in the final five *Bhageerath*-H predictions. The average Molprobity score is 1.94 for 75 predictions.

### *Bhageerath*-H web server

**Figure 8:**
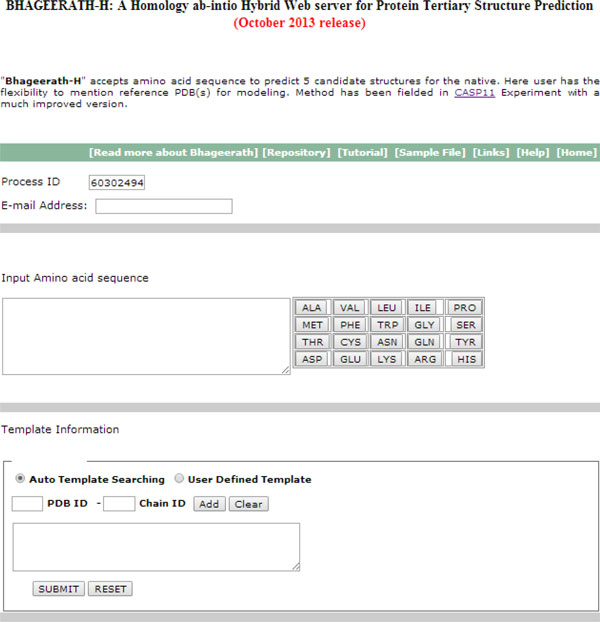
**Screenshot of *Bhageerath*-H web server**.

*Bhageerath*-H automated pipeline is available for the scientific community as a freely accessible web server at url http://www.scfbio-iitd.res.in/bhageerath/bhageerath_h.jsp. The web server takes as input amino acid sequence of the query protein. The processed results are sent to the users at the email id provided by them. Each submitted job is provided with a unique Jobid, which can be used to check job status. The server provides an option for specifying templates. A user can either opt for automatic template searching option or user defined template option. In automatic template searching option software itself searches for the best templates and uses hybrid approach to predict tertiary structure. In user defined template option, user is required to input template information i.e. template's pdb-id and chain id. Structures based on the defined templates will be given to the user as output. Complete *Bhageerath*-H run takes approximately 5-6 hours depending on the size of the protein. The software runs on a 35 node Quad-Core AMD Opteron(tm) Processor 2380 based cluster on CentOS platform over an Infini-band QDR backbone. *Bhageerath*-H receives at least 10-20 jobs every day from all across the world. *Bhageerath*-H is participating in CASP11 competition (1^st ^May 2014 - 16^th ^July 2014). Figure 8 show a screenshot of *Bhageerath*-H webserver.

## Conclusions

We have developed *Bhageerath*-H, an automated pipeline for protein tertiary structure prediction and made it into a freely accessible web server http://www.scfbio-iitd.res.in/bhageerath/bhageerath_h.jsp. The pipeline comprise six different modules which are *Bhageerath*-H Strgen for decoy generation, K-mean clustering, pcSM for decoy selection, PROTSAV for structure validation, QM (PM6) based loop refinement and final ranking. Together each module assists in pushing the prediction accuracy to higher limits. *Bhageerath*-H server was validated on 75 CASP10 targets and results show that the methodology is effective in predicting good structures for proteins with varying sequence and structural similarities. Comparison with some of the existing softwares demonstrated the uniqueness of the hybrid methodology in effectively sampling conformational space, scoring best decoys and refining low resolution models to high and medium resolution. A critical analysis of the targets where *Bhageerath*-H was unsuccessful in predicting low RMSD structures highlights the areas of improvement. These include better secondary structure prediction, better alignment strategies, improvement in *ab initio *modeling for sampling new folds and refinement strategies. We are currently working on these areas especially for targets with very low sequence similarity. The current version of *Bhageerath*-H has already taken the structure prediction field beyond CASP10. This improved methodology is fielded in the ongoing CASP11 experiment.

Several proteins exhibit partial or complete instability in their structures. These proteins are classified as intrinsically disordered proteins (IDPs). *Bhageerath*-H is a homology and *abinito *hybrid method for modeling structures of monomeric proteins. The current web-enabled version of the protocol is not specifically programmed to model structures of IDPs. Rather, the *ab initio *loop modeling section of the first module as well as QM(PM6) method for loop bond angle refinement attempt to sample conformation space of long loop stretches/disordered regions.

Thus to summarize, in the recent years, data driven homology based computational methods have proved successful in predicting tertiary structures for sequences with high sequence similarity. With the dwindling similarities of query sequences, advanced homology/ *ab initio *hybrid approaches are being explored to solve structure prediction problem. Overcoming these limitations while pushing the frontiers of protein structure prediction, we have proposed *Bhageerath*-H algorithm. The proposed algorithm finds applications in the field of protein structure/function prediction, active-site directed drug design, in studying protein-protein interactions, and in protein design and engineering. In the absence of experimental protein structure, the availability of computational protein tertiary structural models helps to probe biological functions of proteins.

## List of abbreviations

CASP: Critical Assessment of Protein Tertiary Structure Prediction.

RMSD: Root mean square deviation.

Cα RMSD: C-alpha root mean square deviation

TM-Score: Template modeling score.

PSPA: Protein structure prediction algorithms.

## Competing interests

The authors declare that they have no competing interests.

## Authors' contributions

PD developed *Bhageerath*-H Strgen, AM developed pcSM, RK and AS developed PROTSAV, PD and GM developed quantum mechanics (PM6) based loop bond angle refinement. BJ supervised the above projects. PD, AM and RK analyzed and validated the server. BJ and PD wrote the manuscript. PD and SS web-enabled the software.

## Supplementary Material

Additional File 1 Cα RMSD and TM-Score of best *Bhageerath*-H prediction. Best refers to lowest Cα RMSD predicted model in final five *Bhageerath*-H predictions. Click here for file

Additional File 2 **Description of biological and structural relevance of CASP10 Targets (T0655, T0672, T0675, T0700, T0716, T0736, T0747, T0755, T0669, T0713, T0686, T0724)**. Click here for file

Additional File 3 Molprobity score of the best *Bhageerath*-H prediction. Click here for file

## Declarations

The publication charges of this article were funded by Professional Development Fund, IIT Delhi, India.

This article has been published as part of *BMC Bioinformatics *Volume 15 Supplement 16, 2014: Thirteenth International Conference on Bioinformatics (InCoB2014): Bioinformatics. The full contents of the supplement are available online at http://www.biomedcentral.com/bmcbioinformatics/supplements/15/S16.
