# Supplementary material for: Bhageerath-H: A homology/ab initio hybrid server for predicting tertiary structures of monomeric soluble proteins
Source: BMC Bioinformatics. 2014 Dec 8;15(Suppl 16):S7. doi: 10.1186/1471-2105-15-S16-S7 (PMC4290660; doi:10.1186/1471-2105-15-S16-S7)
Supplement: Additional File 1 — Cα RMSD and TM-Score of best Bhageerath-H prediction. Best refers to lowest Cα RMSD predicted model in final five Bhageerath-H predictions. [file 1471-2105-15-S16-S7-S1.pdf]

Additional File 1: Cα RMSD and TM-Score of best *Bhageerath*-H prediction. Best refers to lowest Cα RMSD predicted model in final five *Bhageerath*-H predictions.

**S.No.**	**TARGET**	**PDB IDs**	**Cα RMSD (Å) of best *Bhageerath*-H prediction**	**Tm-score of best *Bhageerath*-H prediction**
1	T0644	4fr9	3.3	0.8
2	T0645	4f7a	3	0.9
3	T0649	4f54	13.8	0.4
4	T0650	4fmz	1.4	1
5	T0651	4f67	11.5	0.5
6	T0652	4hg0	8.4	0.6
7	T0653	4fs7	16.3	0.6
8	T0654	4fo5	3.6	0.8
9	T0655	2luz	3.1	1
10	T0657	2lul	3.8	0.8
11	T0658	4fj6	12	0.7
12	T0659	4esn	1.3	0.9
13	T0661	4fcz	3.3	0.8
14	T0662	2lte	2.7	0.8
15	T0663	4exr	10.8	0.5
16	T0664	4f53	2.8	0.9
17	T0666	3ux4	15.5	0.5
18	T0667	4fle	4.4	0.8
19	T0669	2ltl	3	0.8
20	T0671	4fd0	11	0.5
21	T0672	4f0j	5	0.8
22	T0673	4f98	11.2	0.4
23	T0674	4fdy	13.9	0.5
24	T0675	2lv2	5.0	0.5
25	T0676	4e6f	9.2	0.6
26	T0678	4epz	7.7	0.5
27	T0679	4h08	3.9	0.8
28	T0680	4fm3	4.9	0.6
29	T0681	4fxt	2.3	0.9
30	T0682	4jq6	3	0.9

31	T0683	4ezi	2.7	0.9
32	T0684	4gl6	19.1	0.3
33	T0685	4fmt	4.8	0.7
34	T0686	4hqo	7.8	0.6
35	T0687	4hqf	3.8	0.9
36	T0688	4ezg	3.6	0.9
37	T0689	4fvs	1.6	0.9
38	T0690	4gt6	11.9	0.5
39	T0691	4gzv	14	0.5
40	T0692	4h7n	2.7	0.9
41	T0699	4kt7	2.8	0.9
42	T0700	4hfx	3.7	0.5
43	T0703	4hes	3.9	0.9
44	T0704	4hg2	4.3	0.8
45	T0705	4ftd	27.9	0.5
46	T0707	4h41	9.9	0.7
47	T0708	4h17	1	1
48	T0712	4gbs	2.1	0.9
49	T0713	4h09	6.6	0.6
50	T0714	2lvc	1.6	0.9
51	T0715	4c3s	4.8	0.9
52	T0716	2ly9	3	0.7
53	T0717	4h0a	15.1	0.5
54	T0719	4ak1	20.3	0.3
55	T0720	4ic1	16.1	0.6
56	T0721	4fk1	3.1	0.9
57	T0724	4fmr	8.2	0.4
58	T0726	4fgm	10.1	0.6
59	T0731	2lz1	9.4	0.5
60	T0733	4gqa	2.6	0.9
61	T0735	4g2a	17	0.4
62	T0736	4l8p	4.3	0.8
63	T0737	3td7	13.3	0.6
64	T0738	4is2	2	0.9
65	T0742	4ghb	16.2	0.4
66	T0743	4hyz	4.9	0.6
67	T0744	2ymv	11.1	0.5

68	T0746	4gpv	7.9	0.7
69	T0747	4g5a	4.1	0.9
70	T0749	4gl3	1.9	0.9
71	T0752	4gb5	3.6	0.9
72	T0753	4goq	2.6	0.8
73	T0755	4h1x	5	0.8
74	T0756	4g6q	10.5	0.5
75	T0757	4gak	3	0.9
