# Supplementary material for: Bhageerath-H: A homology/ab initio hybrid server for predicting tertiary structures of monomeric soluble proteins
Source: BMC Bioinformatics. 2014 Dec 8;15(Suppl 16):S7. doi: 10.1186/1471-2105-15-S16-S7 (PMC4290660; doi:10.1186/1471-2105-15-S16-S7)
Supplement: Additional File 2 — Description of biological and structural relevance of CASP10 Targets (T0655, T0672, T0675, T0700, T0716, T0736, T0747, T0755, T0669, T0713, T0686, T0724). [file 1471-2105-15-S16-S7-S2.pdf]

Additional File 2: Description of biological and structural relevance of CASP10 Targets (T0655, T0672, T0675, T0700, T0716, T0736, T0747, T0755, T0669, T0713, T0686, T0724)

T0655: Protein Data Bank Id of T0655 is 2LUZ. It is a solution NMR structure of CalU16 from Micromonospora echinospora target MiR12. The protein belongs to the family of eukaryotic, prokaryotic and archaeal proteins that bear similarity to a C-terminal region of human activator of 90 kDa heat shock protein ATPase homolog 1. The protein is an up regulator of HSP90 function and particularly contribute to its efficiency in conditions of increased stress.

T0672: Protein Data Bank Id of T0672 is 4F0J. It is a crystal structure of probable hydrolytic enzyme (PA3053) from Pseudomonas aeruginosa PA01. The protein belongs to alpha/beta hydrolase family and comprise alpha/beta hydrolase fold. The fold contains 8 beta sheets and 6 connected helices which form a barrel structure. It has a catalytic triad a best conserved structural feature of this fold.

T0675: Protein Data Bank Id of T0675 is 2LV2. It is a solution NMR structure of C2H2 -type Zinc-fingers 4 and 5 from human Insulinoma-associated protein 1 (fragment 424-497). The C2H2 zinc finger is the classical zinc finger domain. The two conserved cysteines and histidines co-ordinate a zinc ion. The C2H2 zinc finger is composed of two short beta strands followed by an alpha helix.

T0700: Protein Data Bank Id of T0700 is 4HFX. It is a crystal structure of a transcription elongation factor B polypeptide 3 from Homo sapiens. This protein represents a conserved region within RNA polymerase II transcription factor SIII (Elongin) subunit A. It comprise of 3 helices with a binding site for sulphate ion.

T0716: Protein Data Bank Id of T0716 is 2LY9. It is a Solution NMR Structure of Homeobox 2 Domain from Human ZHX1 repressor. It has a homeobox domain, which is a protein structural domain that binds DNA or RNA. The protein is a transcription factor and

consist of a helix-turn-helix structure motif. The two N-terminal helices are antiparallel and the C-terminal helix is perpendicular to the axes of the first two helices.

T0736: Protein Data Bank Id of T0736 is 4L8P. It is a crystal structure of a bile-acid 7-alpha dehydratase (CLOHIR_00079) from Clostridium hiranonis. This protein comprise of SnoaLfold in which protein adopts a distorted alpha-beta barrel fold.

T0747: Protein Data Bank Id of T0747 is 4G5A. It is a crystal structure of a putative member of duf 3244 protein family (BT_1867) from Bacteroides thetaiotaomicron VPI-5482. The protein adopts an immunoglobulin-like beta-sandwich fold and is structurally similar to fibronectin.

T0755: Protein Data Bank Id of T0755 is 4H1X. It is a Crystal structure of a phosphate ABC transporter, phosphate-binding protein (SP_2084) from Streptococcus pneumoniae TIGR4. The protein belongs to periplasmic binding domain superfamily and consist of a helix-turn-helix domain.

T0669: Protein Data Bank Id of T0669 is 2LTL. It is a solution NMR Structure of NifU-like protein from Saccharomyces cerevisiae. The domain in this protein is commonly found at the N terminal of NifU and NifU related proteins, and in the human Nfu protein. Nifu proteins are involved in the assembly of iron-sulphur clusters.

T0713: Protein Data Bank Id of T0713 is 4H09. It is a crystal structure of a hypothetical leucine rich repeat protein (EUBVEN_01088) from Eubacterium ventriosum ATCC 27560. The protein comprise of a leucine-rich repeat (LLR). LLR is a structural motif that forms a α/β horseshoe fold. Also it consist of 20-30 residue stretches which are rich in leucine.

T0686: Protein Data Bank Id of T0686 is 4HQO. It is a crystal structure of Plasmodium vivax TRAP protein. The protein comprise of a von Willebrand factor domain, which is found in various plasma proteins. The domain consist of a classic alpha/beta Rossmann fold along with an unsual metal ion coordination site at its surface.

T0724: Protein Data Bank Id of T0724 is 4FMR. It is a crystal structure of a hypothetical protein (BVU_2165) from Bacteroides vulgatus ATCC 8482. The protein comprise of a DNA binding domain and domain of unknown function with Immunoglobin like fold.
