# Supplementary material for: Bhageerath-H: A homology/ab initio hybrid server for predicting tertiary structures of monomeric soluble proteins
Source: BMC Bioinformatics. 2014 Dec 8;15(Suppl 16):S7. doi: 10.1186/1471-2105-15-S16-S7 (PMC4290660; doi:10.1186/1471-2105-15-S16-S7)
Supplement: Additional File 3 — Molprobity score of the best Bhageerath-H prediction. [file 1471-2105-15-S16-S7-S3.pdf]

Additional File 3: Molprobity score of the best *Bhageerath*-H prediction

**S.No.**	**Target**	**Molprobity score of best *Bhageerath*-H prediction**
1	T0644	1.8
2	T0645	1.54
3	T0649	2.44
4	T0650	1.31
5	T0651	3.59
6	T0652	1.25
7	T0653	1.82
8	T0654	1.75
9	T0655	1.61
10	T0657	1.46
11	T0658	2.05
12	T0659	1.11
13	T0661	1.07
14	T0662	0.93
15	T0663	2.07
16	T0664	1.64
17	T0666	1.7
18	T0667	1.5
19	T0669	1.38
20	T0671	0.85
21	T0672	1.82
22	T0673	1.44
23	T0674	1.68
24	T0675	1.32
25	T0676	2.2
26	T0678	1.39
27	T0679	2.52
28	T0680	1.59
29	T0681	1.79
30	T0682	1.61
31	T0683	1.65
32	T0684	1.91
33	T0685	2.51
34	T0686	2.27
35	T0687	1.97
36	T0688	1.22
37	T0689	2.53

38	T0690	2.6
39	T0691	1.73
40	T0692	1.56
41	T0699	1.59
42	T0700	1.08
43	T0703	1.67
44	T0704	2.79
45	T0705	2.56
46	T0707	2.66
47	T0708	1.2
48	T0712	1.5
49	T0713	1
50	T0714	1.03
51	T0715	1.83
52	T0716	1.53
53	T0717	2.93
54	T0719	2.74
55	T0720	2.11
56	T0721	1
57	T0724	2.98
58	T0726	2.27
59	T0731	3.12
60	T0733	1.32
61	T0735	2.49
62	T0736	1.65
63	T0737	1.95
64	T0738	0.96
65	T0742	2.53
66	T0743	2.36
67	T0744	2.81
68	T0746	2.05
69	T0747	0.85
70	T0749	1.25
71	T0752	2.12
72	T0753	1.34
73	T0755	3.71
74	T0756	1.85
75	T0757	1.31
**Average**		**1.84**
